# Risk of stroke and heart failure attributable to atrial fibrillation in middle-aged and elderly people: Results from a five-year prospective cohort study of Japanese community dwellers

**DOI:** 10.1016/j.je.2016.08.012

**Published:** 2017-04-05

**Authors:** Masaki Ohsawa, Tomonori Okamura, Kozo Tanno, Kuniaki Ogasawara, Kazuyoshi Itai, Yuki Yonekura, Kazuki Konishi, Shinichi Omama, Naomi Miyamatsu, Tanvir Chowdhury Turin, Yoshihiro Morino, Tomonori Itoh, Toshiyuki Onoda, Kiyomi Sakata, Yasuhiro Ishibashi, Shinji Makita, Motoyuki Nakamura, Fumitaka Tanaka, Toru Kuribayashi, Mutsuko Ohta, Akira Okayama

**Affiliations:** a Department of Internal Medicine, Iwate Medical University, Morioka, Japan; b Department of Internal Medicine, Morioka Tsunagi Onsen Hospital, Morioka, Japan; c Department of Preventive Medicine and Public Health, Keio University, Tokyo, Japan; d Department of Hygiene and Preventive Medicine, Iwate Medical University, Yahaba-cho, Iwate, Japan; e Department of Neurosurgery, Iwate Medical University, Morioka, Japan; f Department of Nutritional Sciences, Morioka University, Takizawa, Iwate, Japan; g Department of Clinical Nursing, Shiga University of Medical Science, Otsu, Japan; h Department of Community Health Sciences, University of Calgary, Calgary, Canada; i Department of Health and Physical Education, Faculty of Education, Iwate University, Morioka, Japan; j Iwate Health Service Association, Morioka, Japan; k The Research Institute of Strategy for Prevention, Tokyo, Japan

**Keywords:** Atrial fibrillation, Stroke, Heart failure, Prospective study, Relative risk, Absolute risk

## Abstract

**Background:**

The relative and absolute risks of stroke and heart failure attributable to atrial fibrillation (AF) have not been sufficiently examined.

**Methods:**

A prospective study of 23,731 community-dwelling Japanese individuals was conducted. Participants were divided into two groups based on the presence or absence of prevalent AF (n = 338 and n = 23,393, respectively). Excess events (EE) due to AF and relative risks (RRs) determined using the non-AF group as the reference for incident stroke and heart failure were estimated using Poisson regression stratified by age groups (middle-aged: 40–69 years old; elderly: 70 years of age or older) after adjustment for sex and age.

**Results:**

There were 611 cases of stroke and 98 cases of heart failure during the observation period (131,088 person-years). AF contributed to a higher risk of stroke both in middle-aged individuals (EE 10.4 per 1000 person-years; RR 4.88; 95% confidence interval [CI], 2.88–8.29) and elderly individuals (EE 18.3 per 1000 person-years; RR 3.05; 95% CI, 2.05–4.54). AF also contributed to a higher risk of heart failure in middle-aged individuals (EE 3.7 per 1000 person-years; RR 8.18; 95% CI, 2.41–27.8) and elderly individuals (EE 15.4 per 1000 person-years; RR 7.82; 95% CI, 4.11–14.9). Results obtained from multivariate-adjusted analysis were similar (stroke: EE 8.9 per 1000 person-years; RR 4.40; 95% CI, 2.57–7.55 in middle-aged and EE 17.4 per 1000 person-years; RR 2.97; 95% CI, 1.99–4.43 in elderly individuals; heart failure: EE 3.1 per 1000 person-years; RR 7.22; 95% CI, 2.06–25.3 in middle-aged and EE 14.1 per 1000 person-years; RR 7.41; 95% CI, 3.86–14.2 in elderly individuals).

**Conclusions:**

AF increased the risk of stroke by the same magnitude as that reported previously in Western countries. AF increased the RR of heart failure more than that in Western populations.

## Highlights

• Incidence rates for stroke and HF were estimated in Japanese community dwellers.
• Subjects were divided into AF and non-AF groups separately by age (40–69 and ≥70).
• AF increased the risk of stroke by the same magnitude as in Western populations.
• AF increased the risk of HF more than that in Western populations.
• The absolute risk differences of outcomes were larger in the elderly individuals.

## Introduction

The most life-threatening sequelae of atrial fibrillation (AF) are stroke and heart failure.^1^ Numerous epidemiological studies have shown that AF is strongly associated with an increased risk of stroke^2^, ^3^, ^4^, ^5^, ^6^, ^7^, ^8^, ^9^ and heart failure,^5^, ^7^, ^9^ as well as all-cause mortality.^5^, ^7^, ^8^, ^9^, ^10^, ^11^, ^12^, ^13^, ^14^, ^15^ Early identification of individuals at high risk of stroke and heart failure will enable us to take preventive measures and implement intervention strategies in order to lessen the burden of AF.

The incidence and prevalence of AF, stroke, and heart failure all greatly increase with age.^16^, ^17^, ^18^, ^19^, ^20^ Age-stratified analysis is necessary to assess the risk of stroke and heart failure attributable to AF. Although age-specific incidences of AF,^16^, ^17^, ^18^ stroke,^19^ and heart failure^20^ have been studied in Western general populations, such data are not available for non-Western populations. The age-specific risks of stroke and heart failure attributable to AF should also be determined in people living in non-Western countries. Thus, we investigated the absolute and relative risks (RRs) of stroke and heart failure both in middle-aged and elderly individuals with and without AF in order to obtain fundamental information on AF-related risks of cardiovascular events in Japan.

## Methods

### Subjects

**Fig. 1:**
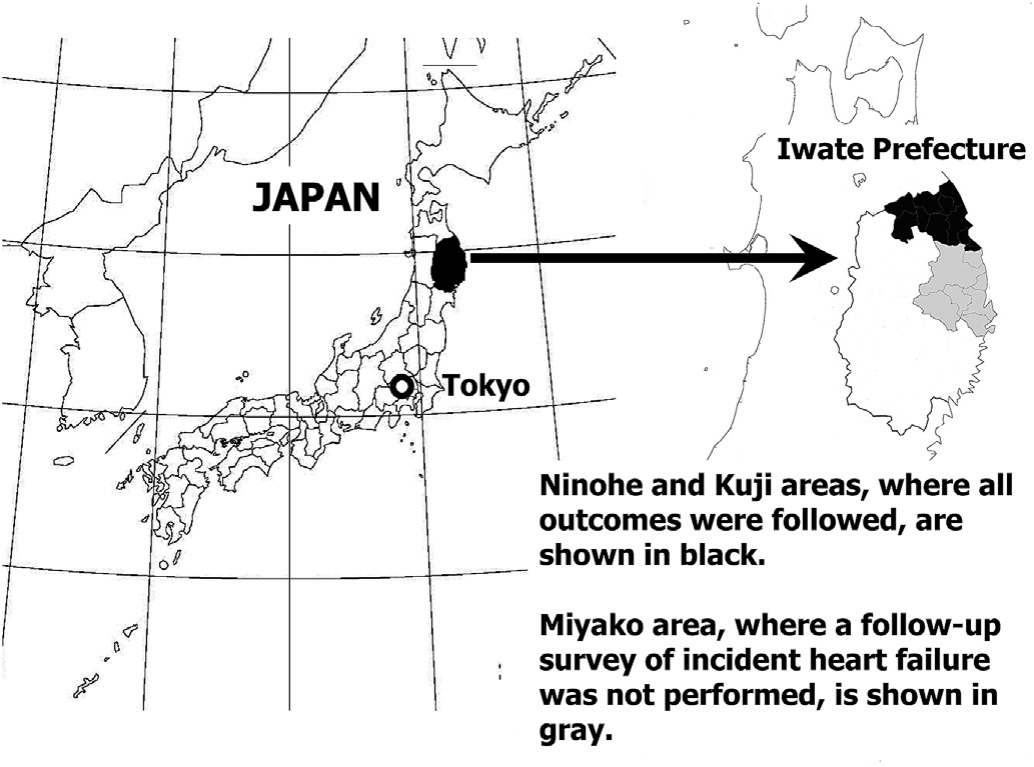
The study area. A map of Japan and Iwate Prefecture. Iwate Prefecture is located in the northeastern part of the main island (Honshu Island) of Japan. Within Iwate Prefecture, the Ninohe and Kuji areas, where all outcomes were followed, are indicated in black. The gray area corresponds to the Miyako area, where a follow-up survey of incident heart failure was not done.

**Fig. 2:**
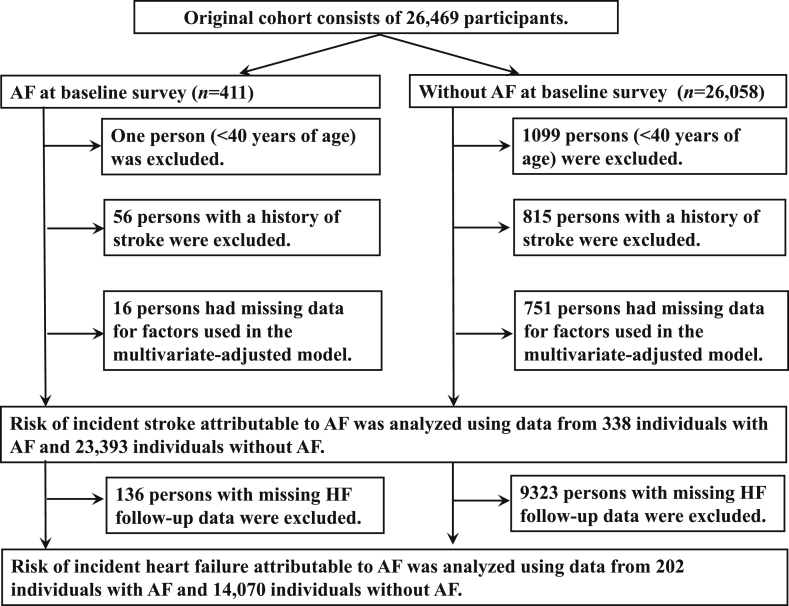
Procedure used to select patients for the Iwate KENCO study. We excluded a total of 2738 subjects. Ultimately, the risk of stroke was analyzed using data from 23,731 subjects and the risk of heart failure was analyzed using data from 14,272 subjects. AF, atrial fibrillation; HF, heart failure.

The study subjects were members of the Iwate-Kenpoku cohort (Iwate-KENCO) study. The study area consists of three local public health center-associated areas (Ninohe, Miyako, and Kuji, shown in Fig. 1). The methodology of the Iwate-KENCO study was described elsewhere.^21^, ^22^ The original study cohort consisted of 26,469 participants. We excluded subjects as shown in Fig. 2. Risk of incident stroke attributable to AF was analyzed using data from 338 individuals with AF and 23,393 individuals without AF. Since data on heart failure were not collected in the Miyako area, we used data for both AF and non-AF participants who lived either in the Ninohe or Kuji area (AF: n = 202; non-AF: n = 14,272) to estimate the risk of incident heart failure attributable to AF (Fig. 1, Fig. 2). The study was approved by the Medical Ethics Committee of Iwate Medical University and conducted in accordance with the guidelines of the Declaration of Helsinki.^21^

### Initial investigation

The initial examination consisted of a questionnaire, measurements of blood pressure and anthropometric data, blood tests, and electrocardiography (ECG). The methods used to determine serum lipid profiles, serum high-sensitivity C-reactive protein levels (hsCRP), plasma glucose levels, plasma glycosylated hemoglobin (HbA_1c_) levels, and urinary albumin:creatinine ratios (UACRs) were described previously.^21^, ^22^, ^23^, ^24^ Twelve-lead ECG was performed in each participant after 5 min of rest. ECG findings were independently evaluated by a trained clinical technician and a medical doctor in the Iwate Health Service Association according to the original coding system. Prevalent cases of AF were determined on the basis of presence of chronic or paroxysmal AF/flutter.^21^, ^25^ A past history of stroke or myocardial infarction was identified through the self-administered questionnaire and using data from the Iwate Prefecture Stroke Registration program^22^, ^26^, ^27^, ^28^, ^29^ and the Northern Iwate Heart Disease Registry Consortium.^30^

### Classification and definition

Participants were divided into a middle-aged group (40–69 years old) and elderly group (≥70 years of age). Overweight was defined as a body mass index (BMI) of 25 kg/m^2^ or higher. Hypertension (HT) was defined as systolic blood pressure (SBP) of 140 mm Hg or higher, diastolic blood pressure (DBP) of 90 mm Hg or higher, use of antihypertensive agents, or a combination thereof. Diabetes mellitus (DM) was defined as plasma glucose level of 200 mg/dL or higher, plasma HbA1c level (National Glycohemoglobin Standardization Program equivalent value) of 6.5% or higher, use of anti-diabetes agents, or a combination thereof among participants who provided non-fasting blood samples (n = 19,934). In participants who provided fasting blood samples (n = 3797), subjects whose fasting glucose level was 126 mg/dL or higher were also included in the DM group, together with subjects with DM determined by the above-described definition. Dyslipidemia was defined as serum total cholesterol (TC) of 220 mg/dL or higher, serum high-density lipoprotein cholesterol (HDLC) level of less than 40 mg/dL, use of anti-hyperlipidemia agents, or a combination thereof. Regular alcohol drinking was defined as consuming alcohol on 5 days or more per week.

### Follow-up surveys

The endpoints were incident stroke, ischemic stroke, and heart failure. The methods used to ascertain the vital status of each participant were described previously.^22^, ^24^, ^26^, ^31^ Stroke events were identified using the Iwate Prefecture Stroke Registration program, which included the entire area where the subjects lived. Details of this registry have been described previously.^22^, ^26^, ^27^, ^28^, ^29^ The medical records of all medical facilities within the survey area were verified every year to ensure complete capture of all data from 2006 to 2009 by physicians and trained research nurses. Incidents of heart failure were identified using data from the Northern Iwate Heart Disease Registry Consortium, which has been collecting data since 2002.

The registration of heart failure was based on the criteria of the Framingham Heart Study.^32^ A definite diagnosis of congestive heart failure requires that a minimum of two major or one major and two minor criteria be present concurrently. Major criteria were: 1) paroxysmal nocturnal dyspnea or orthopnea; 2) distended neck veins; 3) rales; 4) increasing heart size by X-ray; 5) acute pulmonary edema on chest X-ray; 6) ventricular S 3 gallop; 7) increased venous pressure >16 cm H_2_0; 8) hepatojugular reflux; 9) pulmonary edema, visceral congestion, or cardiomegaly shown on autopsy; and 10) weight loss on heart failure therapy. Minor criteria include: 1) bilateral ankle edema, 2) night cough, 3) dyspnea on ordinary exertion, 4) hepatomegaly, 5) pleural effusion by X-ray, 6) decrease in vital capacity by one-third from maximum record, 7) tachycardia (120 beats per minute or more), and 8) pulmonary vascular engorgement on chest X-ray. These criteria were checked using a registration card for hospital inpatients.^30^ To verify the accuracy of the data, physicians and trained research nurses also checked the medical records of the referral hospitals.

### Statistical analysis

Continuous variables were expressed as sex- and age-adjusted means (95% confidence interval [CI]), except for hsCRP and UACR, which were expressed as sex- and age-adjusted geometric means (95% CI) using analysis of covariance (ANCOVA). The sex- and age-adjusted prevalence of each risk factor (overweight, HT, DM, dyslipidemia, current smoking, past smoking, and regular drinking) was estimated using logistic regression analysis. Adjustment for sex was performed with a male:female ratio of 1.0, and age adjustment was performed with a 60-year-old person as the reference in the middle-aged group and a 75-year-old person as the reference in the elderly group, using ANCOVA or logistic regression.

We defined the follow-up period as the period from the initial survey to either the first outcome or the end of the observation period. Individuals who did not experience any outcomes in the follow-up study were administratively censored. The cumulative probability of each outcome was estimated using the Kaplan–Meier method, and differences in the cumulative probability of each outcome were tested using the log-rank test. Crude incidence rates were determined in groups stratified by sex, age, and AF status. Sex- and age-adjusted incidence rates (per 1000 person-years) and rate ratios were determined in each age group using Poisson regression analysis, which was adjusted for a male:female ratio of 1.0. Age adjustment was performed for persons aged 60 years in analysis of the middle-aged group and for persons aged 75 years in the elderly group. Excess events (incident stroke and incident heart failure) per 1000 person-years attributable to AF were determined by subtracting the adjusted incidence rate in subjects without AF from the rate in subjects with AF. Multivariate-adjusted incidence rates and rate ratios were also estimated after adjusting for explanatory variables that were statistically different between the AF and the non-AF groups in ANCOVA or logistic regression analysis. Multivariate adjustment was performed for a person with mean levels of TC, HDLC, HbA_1_c, logarithm-transformed hsCRP, and logarithm-transformed UACR as the references. Sex- and age-adjustment in the multivariate adjustment was performed using the same procedure as that for the sex- and age-adjusted Poisson regression analysis. All *P* values were two-tailed, and values less than 0.05 were considered to be statistically significant. Statistical analyses were performed using the SPSS software package, version 22 (IBM Japan, Tokyo, Japan) and STATA version STATA/SE 11 (Stata Corp, College Station, TX, USA).

## Results

The prevalence of AF in this study was 1.4%. Table 1 shows the baseline characteristics of the AF and non-AF groups in the middle-aged and elderly populations. The mean age was higher in the AF group than in the non-AF group among middle-aged individuals (*P* < 0.05 using Student's t-test). The proportions of male subjects were higher in both the middle-aged and elderly individuals in the AF group (*P* < 0.05 using the chi-squared test). Sex- and age-adjusted mean BMI, HbA1c, hsCRP, and UACR levels were higher in the AF group than in the non-AF group in both middle-aged and elderly individuals (*P* < 0.05 using ANCOVA). The adjusted prevalence of overweight participants in the AF group was higher than that in the non-AF group among middle-aged individuals (*P* < 0.05 using logistic regression). The adjusted prevalence rate of DM was higher in middle-aged individuals in the AF group than in middle-aged individuals in the non-AF group (*P* < 0.05 using logistic regression).

**Table 1:** Baseline characteristics of participants stratified by atrial fibrillation.

Subjects	Middle-aged (40–69 years)		Elderly (70 years or older)	
	Non-AF	AF	Non-AF	AF
	16,697	145	6696	193
Age, years, mean (SD)	58.8 (7.9)	63.0 (5.9)	74.8 (3.7)	75.4 (4.0)
Males, n (%)	5162 (30.9%)	106 (73.1%)	2745 (41.0%)	134 (69.4%)
Sex- and age-adjusted mean (95% CI)				
BMI (kg/m^2^)	24.1 (24.1–24.2)	24.9 (24.4–25.5)*	23.9 (23.8–23.9)	24.7 (24.2–25.1)*
SBP (mmHg)	126.9 (126.6–127.2)	125.7 (122.6–128.8)	133.5 (133.0–134.0)	131.1 (128.4–133.9)
TC (mg/dL)	200.5 (200.0–201.0)	193.1 (187.9–198.4)*	196.9 (196.2–197.7)	190.0 (185.7–194.4)*
HDLC (mg/dL)	59.0 (58.7–59.2)	59.5 (57.1–61.8)	57.5 (57.1–57.8)	58.1 (56.0–60.1)
Hb_A1c_ (%)	5.52 (5.51–5.53)	5.77 (5.66–5.88)*	5.60 (5.58–5.61)	5.79 (5.70–5.88)*
hsCRP^a^ (mg/L)	0.47 (0.46–0.48)	0.63 (0.53–0.75)*	0.57 (0.56–0.59)	0.88 (0.75–1.03)*
UACR^a^ (mg/g)	15.5 (15.3–15.8)	31.9 (27.1–37.6)*	22.7 (22.1–23.3)	38.8 (33.1–45.4)*
Sex- and age-adjusted prevalence (95% CI) expressed as percentage				
Overweight	35.9% (35.2%–36.7%)	44.7% (36.8%–52.9%)†	35.5% (34.4%–36.7%)	43.5% (36.7%–50.6%)
Hypertension	36.6% (35.8%–37.4%)	41.5% (33.7%–49.7%)	58.1% (56.9%–59.3%)	56.3% (49.1%–63.2%)
Diabetes mellitus	6.3% (5.9%–6.7%)	12.3% (8.2%–18.2%)†	9.7% (9.0%–10.4%)	14.3% (10.2%–19.7%)
Dyslipidemia	35.1% (34.3%–35.9%)	33.0% (25.7%–41.1%)	35.6% (34.5%–36.8%)	27.7% (21.7%–34.5%)
Current smoker	9.5% (9.0%–10.1%)	9.6% (6.5%–13.9%)	5.1% (4.7%–5.6%)	3.2% (2.0%–5.1%)
Past smoker	6.3% (5.8%–6.8%)	6.8% (4.6%–9.9%)	8.5% (7.8%–9.3%)	9.0% (6.5%–12.3%)
Regular drinker^b^	17.2% (16.4%–17.9%)	24.9% (18.6%–32.6%)†	10.7% (10.1%–11.6%)	11.4% (8.4%–15.4%)

AF, atrial fibrillation; ANCOVA, analysis of covariance; BMI, body mass index; CI, confidence interval; HbA1c, glycosylated hemoglobin; HDLC, high-density lipoprotein cholesterol level; hsCRP, high-sensitivity C-reactive protein; SBP, systolic blood pressure; TC, total cholesterol level; UACR, urinary albumin-to-creatinine ratio.

Data are expressed as means (standard deviations), sex- and age-adjusted means (95% CI) and sex- and age-adjusted prevalences (95% CI).

Adjusted means were estimated using ANCOVA and adjusted prevalences were estimated using logistic regression after adjusting for age (60 years in persons less than 70 years; 75 years in persons aged 70 years or older) and sex (male:female ratio of 1.0).

^∗^*P* < 0.05 compared to the adjusted mean in non-AF subjects by ANCOVA.

^†^*P* < 0.05 compared to the adjusted prevalence in non-AF subjects by logistic regression analysis.

a Data are expressed as sex- and age-adjusted geometric means (95% CI).

b Regular alcohol drinking was defined as consuming alcohol on 5 days or more per week.

**Fig. 3:**
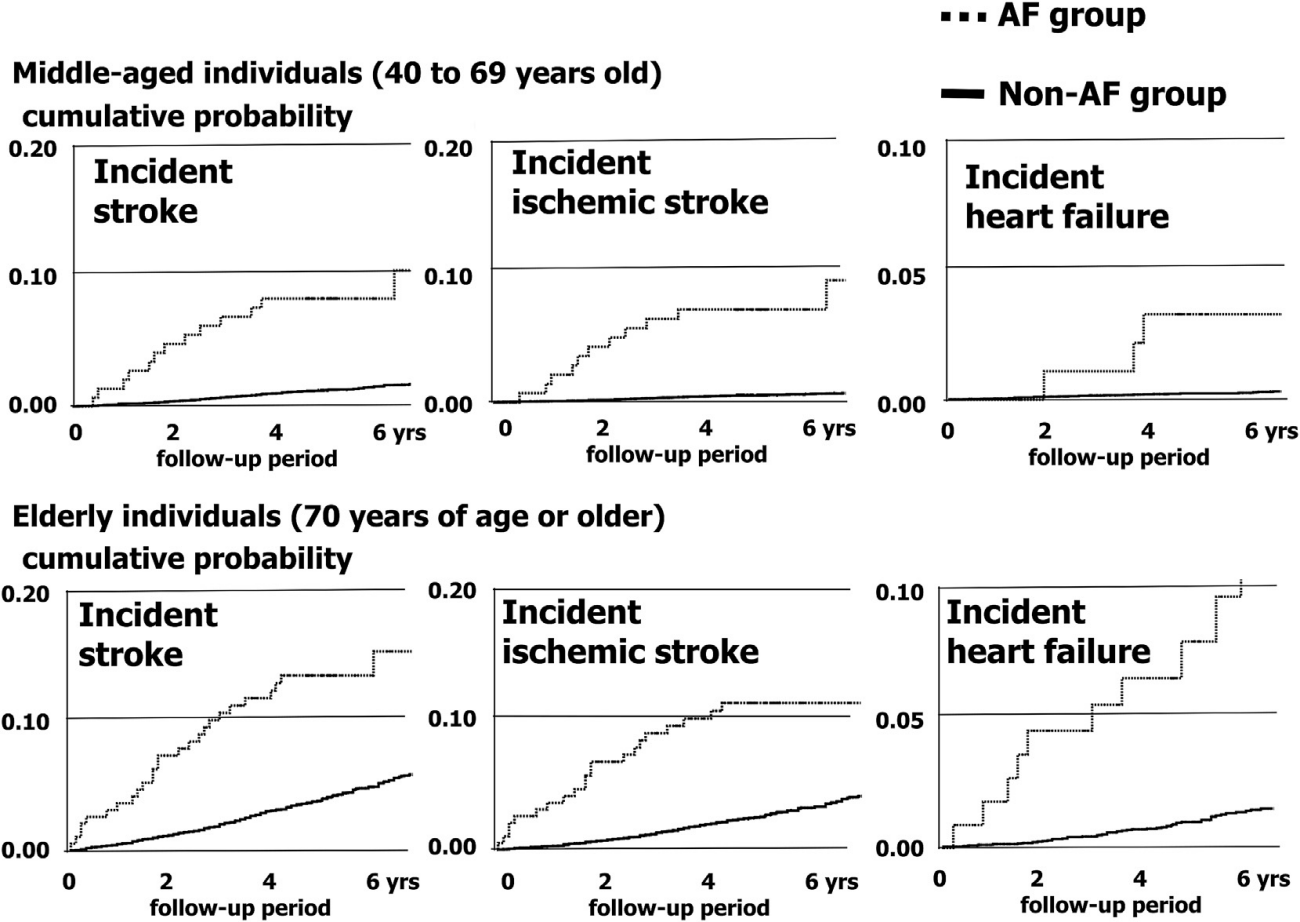
Estimated Kaplan–Meier cumulative probability of incidence in AF and non-AF groups. AF, atrial fibrillation. The upper three graphs show the cumulative probabilities of incident stroke, incident ischemic stroke, and incident heart failure among middle-aged individuals (40–69 years), and the lower three graphs show the cumulative probabilities of these outcomes among elderly individuals (70 years or older). Participants with AF had significantly higher probabilities of these outcomes both in the middle-aged and elderly groups (*P* < 0.001 using the log-rank test). These cumulative probability curves suggest that the incidence rates for the AF group were likely to be proportional to the incidence rates for the non-AF group during the observation period, and it was reasonable to perform Poisson regression analysis to obtain the adjusted relative risk expressed as an adjusted rate ratio.

At the completion of follow up, there were 131,088 observed person-years, and the median follow-up duration was 5.2 years. There were 611 cases of stroke and 98 cases of heart failure during the observation period. Fig. 3 shows the Kaplan-Meier–estimated cumulative probabilities of incidence for participants in the AF and non-AF groups. Both middle-aged and elderly participants with AF had significantly higher cumulative probabilities of incident stroke, ischemic stroke, and incident heart failure (*P* < 0.01 using the log-rank test).

Table 2 shows the incidences and crude incidence rates of stroke, ischemic stroke, and heart failure stratified by age, sex, and AF. Among individuals aged less than 70 years, the crude incidence rate of stroke in female subjects without AF (2.2 per 1000 person-years) was lower than the rate in male subjects (3.8 per 1000 person-years), while the rates were almost same in male and female subjects with AF (about 20 per 1000 person-years). Among individuals aged 70 years or older, the crude incidence rate of stroke in female subjects without AF (7.5 per 1000 person-years) was lower than the rate in male subjects (10.6 per 1000 person-years), whereas the rate in female subjects with AF (39.2 per 1000 person-years) was much higher than the rate in male subjects with AF (26.5 per 1000 person-years).

**Table 2:** Incidences and crude incidence rates stratified by age, sex, and atrial fibrillation

	40–69 years (n = 16,842)		≥70 years (n = 6889)	
**Stroke cohort study (n = 23,731)**				
Combined males and females	non-AF (n = 16,697)	AF (n = 145)	non-AF (n = 6696)	AF (n = 193)
Incident stroke	251 (2.7)	15 (19.8)	317 (8.8)	28 (30.3)
Incident ischemic stroke	107 (1.1)	13 (17.2)	219 (6.1)	23 (24.9)
Males	non-AF (n = 5162)	AF (n = 106)	non-AF (n = 2745)	AF (n = 134)
Incident stroke	110 (3.8)	11 (20.1)	153 (10.6)	17 (26.5)
Incident iscemic stroke	59 (2.1)	10 (18.2)	121 (8.4)	14 (21.8)
Females	non-AF (n = 11,535)	AF (n = 39)	non-AF (n = 3951)	AF (n = 59)
Incident stroke	141 (2.2)	4 (19.2)	164 (7.5)	11 (39.2)
Incident iscemic stroke	47 (0.7)	3 (14.4)	98 (4.5)	9 (32.1)
**Heart failure cohort study (n = 14,272)**				
Combined males and females	non-AF (n = 10,029)	AF (n = 91)	non-AF (n = 4041)	AF (n = 111)
Incident heart failure	29 (0.5)	3 (5.7)	54 (2.3)	12 (20.9)
Males	non-AF (n = 3078)	AF (n = 65)	non-AF (n = 1610)	AF (n = 82)
Incident heart failure	16 (0.9)	2 (5.4)	29 (3.2)	10 (24.1)
Females	non-AF (n = 6951)	AF (n = 26)	non-AF (n = 2431)	AF (n = 29)
Incident heart failure	13 (0.3)	1 (6.6)	25 (1.7)	2 (12.6)

AF, atrial fibrillation.

Data are expressed as number of events (crude incidence rates per 1000 person-years).

Among individuals aged less than 70 years, crude incidence rates of heart failure were 0.3 per 1000 person-years in females and 0.9 per 1000 person-years in males among individuals without AF, and the rates were 6.6 per 1000 person-years in females and 5.4 per 1000 person-years in males with AF. Among individuals aged 70 years or older, the rates were 1.7 per 1000 person-years in females and 3.2 per 1000 person-years in males without AF, and they were 12.6 per 1000 person-years in females and 24.1 per 1000 person-years in males with AF.

Table 3 shows the sex- and age-adjusted incidence rate and the multivariate-adjusted incidence rate, as well as the adjusted RR for each outcome. Sex- and age-adjusted analyses showed that AF contributed to 10.4 excess strokes per 1000 person-years in the middle-aged group (RR 4.88) and 18.3 excess strokes per 1000 person-years in the elderly group (RR 3.05). AF also contributed to 7.8 excess ischemic strokes per 1000 person-years in the middle-aged group (RR 8.23) and 14.3 excess ischemic strokes per 1000 person-years in the elderly group (RR 3.36). AF contributed to 3.7 excess heart failures per 1000 person-years in the middle-aged group (RR 8.18) and 15.4 excess heart failures per 1000 person-years in the elderly group (RR 7.82).

**Table 3:** Sex- and age-adjusted incidence rate and multivariate-adjusted incidence rate and adjusted relative risk for each outcome by age group

Outcomes		Age groups	Adjusted incidence rate (95% CI)		Excess events	Adjusted RR (95% CI)
			Non-AF	AF		
Stroke	Sex- and age-adjusted	40–69 years	2.68 (2.34–3.07)	13.1 (7.99–22.0)	10.4	4.88 (2.88–8.29)
		≥70 years	8.89 (7.96–9.93)	27.1 (18.5–39.7)	18.3	3.05 (2.05–4.54)
	Multivariate-adjusted	40–69 years	2.63 (2.29–3.01)	11.6 (6.80–19.7)	8.9	4.40 (2.57–7.55)
		≥70 years	8.85 (87.92–9.89)	26.2 (17.9–38.6)	17.4	2.97 (1.99–4.43)
Ischemic stroke	Sex- and age-adjusted	40–69 years	1.08 (0.86–1.34)	8.87 (4.96–15.8)	7.8	8.23 (4.57–14.8)
		≥70 years	6.07 (5.31–6.95)	20.4 (13.3–31.2)	14.3	3.36 (2.15–5.23)
	Multivariate-adjusted	40–69 years	1.04 (0.83–1.31)	8.03 (4.45–14.5)	7.0	7.69 (4.25–13.9)
		≥70 years	6.02 (5.26–6.89)	19.5 (12.6–30.0)	13.4	3.23 (2.07–5.06)
Heart failure	Sex- and age-adjusted	40–69 years	0.52 (0.36–0.75)	4.23 (1.31–13.6)	3.7	8.18 (2.41–27.8)
		≥70 years	2.26 (1.72–2.97)	17.7 (9.80–31.9)	15.4	7.82 (4.11–14.9)
	Multivariate-adjusted	40–69 years	0.50 (0.35–0.73)	3.64 (1.09–12.2)	3.1	7.22 (2.06–25.3)
		≥70 years	2.21 (1.67–2.91)	16.4 (8.94–29.9)	14.1	7.41 (3.86–14.2)

AF, atrial fibrillation; CI, confidence interval; RR, relative risk

Adjusted incidence rates and excess events are expressed per 1000 person-years.

Adjusted incidence rates and relative risks were estimated using Poisson regression after adjustment for age (60 year in persons aged less than 70 years; 75 years in persons aged 70 years or older), sex (male:female ratio of 1.0), TC (mean), HbA1c (mean), hsCRP (mean), and UACR (mean).

Multivariate-adjusted analyses showed 0.6- to 1.5-times lower excess events per 1000 person-years and 0.09- to 0.96-times lower RR than those in the sex- and age-adjusted analyses. Although all excess events and relative risks attributable to AF were mildly decreased after the multivariable adjustment, similar results were obtained in the sex- and age-adjusted model and the multivariable-adjusted model.

## Discussion

Our results showed that adjusted incidence rates and adjusted rate ratios based on Poisson regression were significantly higher for incident stroke, incident ischemic stroke, and incident heart failure in the AF group than in the non-AF group. AF increased the RRs of incident stroke, incident ischemic stroke, and incident heart failure by four to five times, seven to eight times, and seven to eight times, respectively, in the middle-aged group, and by three times, three times, and seven to eight times, respectively, in the elderly group. The absolute risk differences of outcomes expressed as excess events attributable to AF were larger in the elderly individuals than in the middle-aged individuals. Thus, AF contributed to a higher risk of stroke and heart failure in Japanese individuals, as was previously demonstrated in individuals in Western countries.^2^, ^3^, ^4^, ^5^, ^7^, ^8^, ^9^

Previous studies showed that AF increased the RR of incident stroke by two to five times.^2^, ^3^, ^5^, ^7^, ^8^, ^32^, ^33^ Our data showed that AF increased the RR of stroke by five times in middle-aged individuals and by three times in the elderly. The RRs obtained in our study were consistent with those in previous reports. There have been few studies in which the absolute risk of stroke in a general population was investigated. The Framingham Heart Study showed age-specific incidence rates of stroke of 4.1 per 1000 person-years (50–59 years of age), 9.0 per 1000 person-years (60–69 years), 18.0 per 1000 person-years (70–79 years), and 28.7 per 1000 person-years (80–89 years) in individuals without AF and corresponding rates of 55.0 per 1000 person-years, 41.5 per 1000 person-years, 97.9 per 1000 person-years, and 142.9 per 1000 person-years in individuals with AF.^2^ The age-specific incidence rates in subjects with AF and those without AF in our study were two to three times lower than the corresponding rates in the Framingham Study. On the other hand, the Hisayama Study showed that crude incidence rates of ischemic stroke in Japanese community dwellers were 6.4 per 1000 person-years in men and 3.4 per 1000 person-years in women.^6^ Although that report did not show the absolute risk of ischemic stroke among individuals with AF, the RR of ischemic stroke attributable to AF was 3.7,^6^ which is consistent with the RR in the combined middle-aged and elderly groups in our study.

The REACH registry (n = 44,518; mean age, 68.4 years) showed that multivariate-adjusted incidence rates of stroke were 10 per 1000 person-years in the non-AF group and 19 per 1000 person-years in the AF group.^34^ The J-RHYTHM Registry (patients with non-valvular AF: n = 7406) showed that the crude incidence rate of stroke in patients with AF was 14 per 1000 person-years.^35^ These recent outpatient studies showed incidence rates of stroke similar to that in our study in the AF group. However, the incidence rate of stroke in community dwellers, especially in non-AF subjects, was not determined in any previously studies in Japan, and further investigations of the risk of stroke attributable to AF should thus be conducted on the basis of data from the Japanese general population.

Studies on the risk of heart failure attributable to AF have not been conducted in non-Western countries. Previous studies in Western countries showed that AF increased the RR of incident heart failure by 2.35–3.4 times.^5^, ^7^, ^9^ Our data showed that AF increased the RR of heart failure by 8 times in middle-aged and elderly individuals. The RRs obtained in our study were higher than those in the previous studies conducted in the United States.

The reason why the RR of heart failure due to AF was higher in our study than in previous studies might be due to the great difference between incidences of heart failure in Western and Japanese people. Among Medicare beneficiaries, age-specific incidence rates of heart failure from 1994 to 2003 were as high as 20 per 1000 person-years in persons aged 65–69 years, in the low 20 per 1000 person-years in persons aged 70–74 years, approximately 35 per 1000 person-years in persons aged 75–79 years, and approximately 50 per 1000 person-years in persons aged 80–84 years.^20^ On the other hand, the age-specific incidence rates of heart failure in the non-AF group in our study were 0.49 per 1000 person-years in middle-aged individuals and 2.22 per 1000 person-years in those aged 70 years or older. Medicare beneficiaries living in the United States have a 15- to 40-times higher age-specific incidence of heart failure than the incidence rate in our study. A very low incidence rate of heart failure in individuals without AF contributes to a much lower denominator value and thus perhaps to an increase in the RR of heart failure due to AF. Furthermore, Japanese individuals have a very low incidence rate of coronary artery disease (CAD)^36^ and may have a lower incidence rate of CAD-related heart failure and a higher incidence rate of heart failure related to other causes than those in individuals in Western countries. A relatively higher proportion of non-CAD-related heart failure also might contribute to the higher RR of heart failure due to AF among Japanese individuals.

Table 2 shows that AF contributes to the higher risks of stroke and heart failure in female individuals than those in male individuals. Previous studies also indicated that AF contributes to the higher risk of stroke in female subjects.^37^, ^38^, ^39^ Although several reasons, such as sex-based differences in characteristics of coagulation factors and left atrial structure, might contribute to the difference, the mechanism behind the observed difference was unclear.^39^ We also showed a higher absolute risk of heart failure in female subjects with AF. Only one previous study has addressed this issue. Stewart et al showed identical relative risks of heart failure attributable to AF in men and women^7^; however, whether a sex-based difference in the absolute and relative risks of heart failure attributable to AF exists has not been sufficiently examined, and we cannot draw a conclusion yet. Further studies on this issue should be conducted.

### Limitations

This study has several limitations. Risk factors were only determined once, at baseline. Because AF was determined on the basis of routine examinations of 12-lead ECGs recorded by Iwate Health Service Association, some cases with paroxysmal AF were not included. Assessment of the relative risk of outcomes due to prevalent AF does not take into account the subsequent development of AF, which might have led to underestimation of the relative risk. Results based on a multivariate-adjusted model might be distorted by insufficient events per variable.^40^ However, the results based on the multivariate-adjusted model were almost the same as the results based on the sex- and age-adjusted model, so we believe that results based on the multivariate-adjusted model reflected the true RR of outcomes attributable to AF. We observed higher incidence rates of hemorrhagic stroke in the AF group among elderly individuals. The use of an anti-coagulant might have contributed to the higher incidence rate of stroke; however, we do not have information on the use of anti-coagulant therapy, which is one of the limitations of this study.

The present report is the first report on the absolute risk of heart failure in individuals with AF in a Japanese general population; however, the number of incident heart failures was not sufficient for statistical analysis, especially in middle-aged individuals with or without AF and elderly individuals with AF. The estimated risk of heart failure attributable to AF obtained in our study was not thought to be robust. Further studies should be conducted to determine the risk of heart failure attributable to AF in the Japanese general population, and the RR in our study should be re-evaluated by comparison with the results of these future studies.

In conclusion, adjusted rates for incident stroke, incident ischemic stroke, and incident heart failure in the AF group were higher than those in the non-AF group. AF increased the risk of stroke by the same magnitude as that determined in previous studies in Western countries. AF increased the risk of heart failure more than that in Western populations.

## Conflicts of interest

None declared.
